# Cytoglobosins H and I, New Antiproliferative Cytochalasans from Deep-Sea-Derived Fungus *Chaetomium globosum*

**DOI:** 10.3390/md14120233

**Published:** 2016-12-20

**Authors:** Zhihan Zhang, Xitian Min, Junjun Huang, Yue Zhong, Yuehua Wu, Xiaoxia Li, Yinyue Deng, Zide Jiang, Zongze Shao, Lianhui Zhang, Fei He

**Affiliations:** 1 Integrative Microbiology Research Centre, College of Agriculture, South China Agricultural University, Guangzhou 510642, China; zhihanzhang1992@163.com (Z.Z.); mixmix@stu.scau.edu.cn (X.M.); sloe96824400@126.com (Y.Z.); wuyuehua_10@163.com (Y.W.); m18320728922@163.com (X.L.); ydeng@scau.edu.cn (Y.D.); zdjiang@scau.edu.cn (Z.J.); lhzhang01@scau.edu.cn (L.Z.); 2 Pharmaceutical Research Center, School of Pharmacology, Guangzhou Medical University, Guangzhou 510182, China; huangjunjun1985@sina.com; 3 Guangdong Innovative and Entrepreneurial Research Team of Sociomicrobiology Basic Science and Frontier Technology, South China Agricultural University, Guangzhou 510642, China; 4 State Key Laboratory Breeding Base of Marine Genetic Resources, Third Institute of Oceanography, State Oceanic Administration, Key Laboratory of Marine Genetic Resources of Fujian Province, Xiamen 361005, China; shaozz@163.com

**Keywords:** cytochalasans, fungus, antiproliferative activity

## Abstract

Cytoglobosins H (**1**) and I (**2**), together with seven known cytochalasan alkaloids (**3**–**9**), were isolated from the deep-sea-derived fungus *Chaetomium globosum*. The structures of new compounds **1** and **2** were elucidated by extensive 1D and 2D NMR and mass spectroscopic data. All the compounds were evaluated for their antiproliferative activities against MDA-MB-231 human breast cancer cells, LNCaP human prostate cancer cells, and B16F10 mouse melanoma cells. Compound **6** showed significant antiproliferative activity against LNCaP and B16F10 cell lines with IC_50_ values of 0.62 and 2.78 μM, respectively. Further testing confirmed that compound **6** inhibited the growth of LNCaP cells by inducing apoptosis.

## 1. Introduction

Marine microbes have been recognized as a rich source of pharmacologically active metabolites, and a growing number of marine fungi have been reported to produce metabolites with unique structures and interesting biological activities [1]. Furthermore, secondary metabolites from deep-sea fungus, with antimicrobial, antifungal, cytotoxic, antiviral, and antiprotozoal activities, have been well studied during recent decades [2,3]. The genus *Chaetomium*, which includes both terrestrial- and marine-derived species, is well-known for producing structurally complex natural products with antioxidant, antimicrobial, and cytotoxic bioactivities [4,5,6,7,8]. Cytochalasans are an important class of fungal alkaloids with a wide range of structural diversity and biological activities; they have been an important chemical tool in cell and molecular biology and also represent an ambitious target for total synthesis. Biogenetically, the fungal PKS-NRPS (polyketide synthase and non-ribosomal polypeptide synthase) hybrid synthase CheA plays an important role in chaetoglobosin formation [9,10].

In our continuing search for novel bioactive natural compounds from marine fungi [11,12,13,14], we investigated the chemical constituents of *Chaetomium globosum*, a fungus obtained from a deep-sea sediment sample (−2500 m depth) of the Indian Ocean. Two new compounds, cytoglobosins H (**1**), and I (**2**), together with seven known ones, cytoglobosin B (**3**) [8], chaetoglobosinF_ex_ (**4**) [15], chaetoglobosin F (**5**) [16], chaetoglobosin E (**6**) [17], cytoglobosin C (**7**) [8], chaetoglobosin B (**8**) [16], and isochaetoglobosin D (**9**) [18], were isolated. All the compounds were evaluated for their antiproliferative activity towards MDA-MB-231 human breast cancer cells, LNCaP human prostate cancer cells, and B16F10 mouse melanoma cells. Compound **6** showed significant antiproliferative activity on LNCaP and B16F10 cell lines with IC_50_ values of 0.62 and 2.78 μM, respectively. In addition, compound **6** inhibited the growth of LNCaP cells by inducing apoptosis.

## 2. Results and Discussion

### 2.1. Purification and Structure Elucidation of Compounds ***1**–**9***

**Figure 1:**
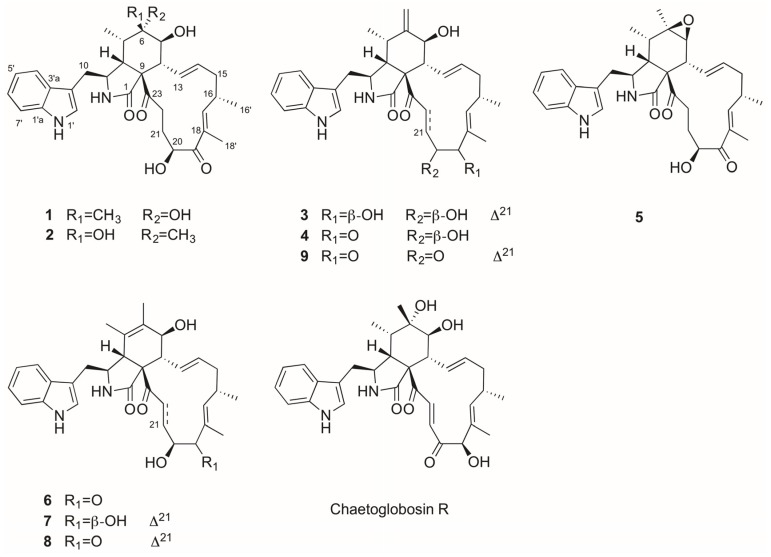
Structure of compounds **1**–**9**.

Repeated flash column chromatography and semi-preparative HPLC of EtOAc extract of *Chaetomium globosum* led to the isolation of nine cytoglobosins, including two new compounds, cytoglobosins H (**1**), and I (**2**), together with seven known ones, cytoglobosin B (**3**) [8], chaetoglobosinF_ex_(**4**) [15], chaetoglobosin F (**5**) [16], chaetoglobosin E (**6**) [17], cytoglobosin C (**7**) [8], chaetoglobosin B (**8**) [16], and isochaetoglobosin D (**9**) (Figure 1). All the known compounds were identified by extensive study of their ^1^H NMR, ^13^C NMR, and ESIMS data, as well as by comparison with those reported in the literature.

**Figure 2:**
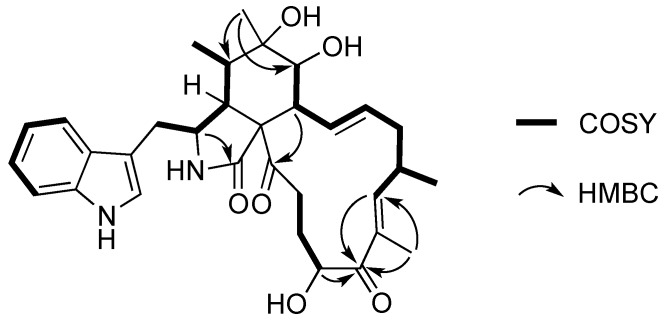
Key COSY and HMBC correlations of compound **1**.

**Figure 3:**
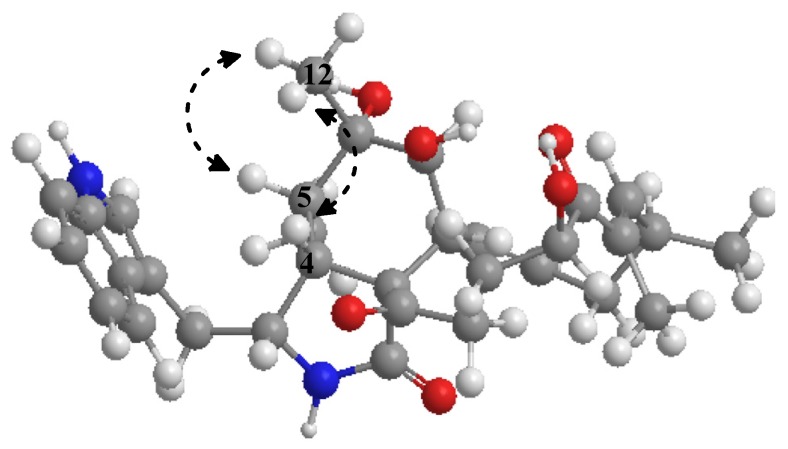
Key ROESY correlations of compound **1**.

Compound **1** was obtained as a white amorphous powder with molecular formula C_32_H_40_N_2_O_6_ through an analysis of its HRESIMS (549.2951, calcd. 549.2959), requiring 14 degrees of unsaturation. In the ^1^H NMR spectrum of **1** (Table 1), the characteristic protons for four methyl groups (*δ*_H_ 1.79, s; 1.24, s; 1.05, d, *J* = 7.6 Hz; and 1.03, d, *J* = 6.6 Hz) were observed. The ^1^H and ^13^C NMR spectra (Table 1) revealed the presence of four methyl groups, four methylene groups, 15 methine groups (including two oxygenated methine carbons and eight olefinic carbons), and nine quaternary carbons, which were quite similar to those of chaetoglobosin R [4]. The positions of two carbonyl groups (*δ*_C_ 205.9, C-19; 209.8, C-23) were determined by HMBC correlations from H-17 (*δ*_H_ 6.24, d) and CH_3_-18 (*δ*_H_ 1.78, s) to C-19, and from H-8 (*δ*_H_ 2.32, m) to C-23. The hydroxyl group on C-19 (*δ*_C_ 81.4) in chaetoglobosin R was positioned on C-20 (*δ*_C_ 72.3) in **1**, which was confirmed by COSY correlations between H_2_-22 (*δ*_H_ 1.65 and 2.38)/H_2_-21(*δ*_H_ 1.60 and 1.37)/H-20 (*δ*_H_ 4.75) (Figure 2). Additionally, the chemical shifts of C-19 (*δ*_C_ 205.9) and C-20 (*δ*_C_ 72.3) combined with the ^1^H-^1^H COSY correlation between H-20 (*δ*_H_ 4.75) and H_2_-21 (*δ*_H_ 1.65 and 2.38) and the HMBC correlation from C-18′ to C-19 indicated that the position of the carbonyl and the oxygenated methine were reversed compared to that of chaetoglobosin R. Further analysis of combined 1D and 2D NMR spectra revealed the difference of relative stereochemistry on C-6 between **1** and chaetoglobosin R. In ROESY spectrum, strong correlations between H_3_-12 and H-4/H-8, between H-8 and H-5, and between H-3 and H_3_-11/H-7 indicated the β-orientation of H-4/H-5/H-8/CH_3_-12 and *α*-orientation of H-3/H-7 (Figure 3). Furthermore, the β-orientation of 20-OH was determined by ROESY correlations between H-17 and H-20/H-15α, between H-13 and H-15α/H-7. Additional supporting evidences for the structure of compound **1** were provided by HMBC and ROESY spectra (Supplementary Materials), which allowed the confirmation of the relative stereochemistry of compound **1**.

Compound **2** was obtained as a white powder, for which the molecular formula was assigned as C_32_H_40_N_2_O_6_ on the basis of HRESIMS, from a [M + H]^+^ ion at *m*/*z* 549.2943 (calcd. 549.2959). On analysis of its ^1^H and ^13^C NMR spectra, similar features to **1** were evident. However, the oxygenated carbons, including one methine (*δ*_C_ 73.6, C-7) and one quaternary carbon (*δ*_C_ 74.1, C-6), were quite different to those in **1** (*δ*_C_ 76.7, C-7; 77.4, C-6), indicating the differences of relative stereochemistry on C-6. Furthermore, correlations between H_3_-11/H_3_-12 and H_3_-12/H-7α in the ROESY spectrum confirmed the α-orientation of methyl group and β-orientation of hydroxyl group on C-6, which established that compound **2** was the C-6 epimer of compound **1**.

Known compounds **3**–**9** were identified as cytoglobosin B (**3**), chaetoglobosinF_ex_ (**4**), chaetoglobosin F (**5**), chaetoglobosin E (**6**), cytoglobosin C (**7**), chaetoglobosin B (**8**), and isochaetoglobosin D (**9**), respectively, by comparison of their MS and NMR data with those reported in the literature.

### 2.2. Antiproliferative Assay of Compounds ***1**–**9***

**Figure 4:**
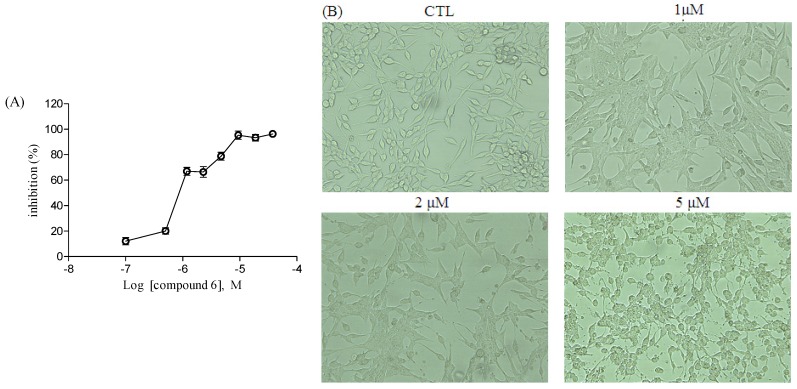
Effects of compound **6** on LNCaP cell proliferative inhibition. (**A**) Effect of compound **6** on cell proliferation inhibition against LNCaP cell lines. The cell proliferation inhibition (%) of three cancer cells after treatment with different concentrations of **6** for 48 h was measured using WST-8 cell proliferation assay; (**B**) Photomicrograph of the LNCaP cells visualized (100× magnification) using Nikon inverted phase contrast microscope. Concentration- and time-dependent effects were observed. Each value represents the mean ± S.E.M. of threeindependent experiments.

The antiproliferative bioactivities of compounds **1**–**9** towards MDA-MB-231 human breast cancer cells, LNCaP human prostate cancer cells, and B16F10 mouse melanoma cells were evaluated, and the IC_50_ values are shown in Table 2. MDA-MB-231 cells were resistant to tested compounds with no obvious cytotoxicity (IC_50_ > 10 μM). Most of them showed potent antiproliferative activity on LNCaP and B16F10 cells (IC_50_ < 10 μM) expect compound **2**. In LNCaP cells, compounds **1** and **4**–**9** possessed considerable antiproliferative effects with IC_50_ ranging from 0.62 μM to 7.78 μM. In B16F10 cells, compounds **3**–**8** possessed considerable antiproliferative effects, with IC_50_ ranging from 2.10 μM to 7.15 μM. Compound **6** showed the highest cytotoxic activity and displayed a promising antitumor activity against the LNCaP and B16F10 cell lines, with IC_50_ values of 0.62 and 2.78 μM, respectively, similar to the positive control cisplatin (Figure 4).

**Figure 5:**
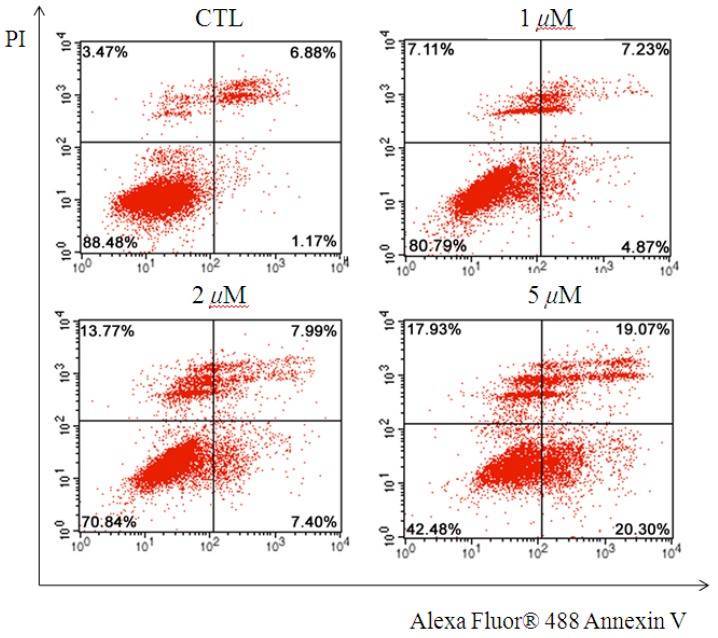
Externalization of phosphatidylserine in LNCaP treated with **6** (0, 1, 2, and 5 μM) for 24 h was detected through Alexa Fluor^®^ 488 Annexin V/PI double staining assay. The cell population in the lower right quadrant (Annexin V^+^/PI^−^) represents early apoptotic cells, whereas the population in the upper right quadrant (Annexin V^+^/PI^−^) represents late apoptotic cells or dead cells.

Further experiments were conducted to determine whether or not the antiproliferative effect of compound **6** on LNCaP cell viability is closely associated with apoptosis. Quantification by Alexa Fluor^®^ 488 Annexin V/PI double staining assay showed that **6** increased the percentage of apoptotic cells in a dose-dependent manner in LNCaP cells (Figure 5). Treatment with **6** for 24 h significantly increased the number of apoptotic LNCaP cells at both early- and late-stage apoptosis. Statistical analysis indicated that the apoptotic rates were approximately 12.10%, 15.39%, and 39.36% after treatment with compound **6** at 1.0, 2.0, and 5.0 μM, respectively. Investigations into the mechanistic aspects of the antigrowth effect of **6** against prostatic tumors are currently in progress.

## 3. Experimental Section

### 3.1. General Experimental Procedures

Optical rotations were determined with an MCP 300 (Anton Paar) polarimeter at 25 °C. UV spectra were recorded on a U-2910 spectrometer (Hitachi, Kyoto, Japan). IR spectra were recorded on Affinity-1 FT-IR spectrometer (Shimadzu, Kyoto, Japan). NMR spectra were recorded with an Avance 500 spectrometer (Bruker, Bremen, Germany) at 500 MHz for ^1^H nucleus and 125 MHz for ^13^C nucleus. Chemical shifts (δ) are given with reference to TMS. Coupling constants (*J*) are given in Hz. ESIMS spectra were detected with an Esquire 3000 plus spectrometer (Bruker). HRESIMS data were acquired using a micro TOF-QII mass spectrometer (Bruker). Column chromatography (CC) was performed using a silica gel (100–200 mesh; Qingdao Marine Chemicals, Qingdao, China) and Sephadex LH-20 (Amersham Pharmacia, Uppsala, Sweden). HPLC was carried out on an ODS column (4.6 × 250 mm, 5 μm; 10 × 250 mm, 5 μm, Phenomenex, Los Angeles, CA, USA) with a photodiode detector (AgileSnt 1260, Santa Clara, CA, USA).

### 3.2. Fungal Materials

The fungal strain of *C. globosum* MCCC 3A00607 was isolated from deep-sea sediments collected from the Indian Ocean. The strain was identified by Dr. Zhongze Shao, and a voucher specimen (*C. globosum* MCCC 3A00607) has been deposited in the Marine Culture Collection of China.

### 3.3. Fermentation and Isolation

The fungal strain of *C. globosum* MCCC 3A00607 was cultured in a rice medium at 28 °C without shaking for 30 days. The fermented solid rice medium (2 kg) was extracted with 70% acetone/water, and evaporated under reduced pressure to afford an aqueous solution, which was then extracted three times with EtOAc and afforded the EtOAc extract (11 g). The EtOAc-soluble material (10 g) was chromatographed on normal silica gel using gradient elution from 100% (*v*/*v*) CHCl_3_ to 100% MeOH (*v*/*v*), to give six crude fractions (Fr. C1–Fr. C6). Fr. C1 eluted with 100% CHCl_3_, containing mostly non-polar constituents such as fatty acid and sterols, was not further investigated. Fraction C2 (CHCl_3_/MeOH, 95:5 *v*/*v* elution, 3.8 g) was further purified by flash column chromatography (gradient elution from 98% (*v*/*v*) CHCl_3_/MeOH to 50% CHCl_3_/MeOH (*v*/*v*), 80 g silica column, 30 min/mL) to afford eight sub-fractions, Fr. C2-1–Fr. C2-8. Compound **5** (60 mg) was crystallized from a mother liquid of Fr. C2-2 (97:3, CHCl_3_/MeOH, 1.26 g). Fr. C2-2 was further purified by semi-preparative reversed-phase HPLC (ACN/H_2_O 45% *v*/*v*, 3 mL/min, UV detector 220 nm) to afford compounds **6** (20 mg), **8** (14 mg), and **9** (6 mg). Fr. C2-3 (0.9 g) was subjected to sephedex-LH20 (CHCl_3_/MeOH, 1:1, *v*/*v*) to obtain five fractions (Fr. C2-3-1–Fr. C2-3-5). Compounds **2** (10 mg) and **4** (6 mg) were isolated from Fr. C2-3 (95:5, CHCl_3_/MeOH) by semi-preparative HPLC (3 mL/min, 50% MeOH/water, UV detector 220 nm) with retention times of 22.5 min and 14.9 min, respectively. Fr. C2-4 (0.68 g) was further purified by semi-preparative reversed-phase HPLC (MeOH/H_2_O 60% *v*/*v*, 3 mL/min, UV detector 220 nm) to afford compounds **1** (8 mg), **3** (2 mg), and **7** (6 mg).

Cytoglobosins H (**1**): white amorphous powder; [*α*]${}_{D}^{25}$ −50.4° (*c* 0.01, CH_3_OH); UV (CH_3_OH) λ_max_ (log ε) 220 (4.06); IR υ_max_ (cm^−1^) 3294, 2956, 1651, 1429, 1024; ^1^H and ^13^C NMR data see Table 1; HRESIMS *m*/*z* 549.2951 [M + H]^+^ (calcd. for C_32_H_41_N_2_O_6_, 549.2959).

Cytoglobosins I (**2**): white amorphous powder; [*α*]${}_{D}^{25}$ −22.1° (*c* 0.01, CH_3_OH); UV (CH_3_OH) λ_max_ (log ε) 221 (3.98); IR υ_max_ (cm^−1^) 3278, 2922, 1668, 1456, 1016; ^1^H and ^13^C NMR data see Table 1; HRESIMS *m*/*z* 549.2943 [M + H]^+^ (calcd. for C_32_H_41_N_2_O_6_, 549.2959).

### 3.4. Antiproliferative Activity of Compounds ***1**–**9***

We examined the effect of compounds **1**–**9** on the proliferation of the MDA-MB-231, LNCaP and B16F10 cell lines in vitro. Each cell line was seeded in 96-well plates at 1 × 10^4^ cells/well in 100 μL culture medium. After seeding for 24 h, the medium was removed and replaced with a fresh medium containing the same concentration of DMSO (1%) as a vehicle control or with a medium containing increased concentrations of the target compounds (from 0 to 20 μM) in a final volume of 100 μL. The culture was maintained in a CO_2_ incubator for an additional 48 h. After 48 h, 10 μL/well Cell Proliferation Reagent CCK-8 (Dojindo Molecular Technologies, Kyushu, Japan) was added and incubated for 1 h at 37% and 5% CO_2_. The absorbance of the formed formazan product was detected at 450 nm in a 96-cell spectrophotometric plate reader following the manufacturer’s instructions. The concentration required to inhibit cell growth by 50% (IC_50_) was calculated from inhibition curves.

### 3.5. Apoptosis Evaluation of Compound ***6***

LNCaP cells were seeded in six-well plates and then cultured for 24 h. The cells were treated with different concentrations of **6** (0, 1, 2, 5, 10, and 20 μM) for 24 h, collected, and stained using Alexa Fluor^®^ 488 Annexin V/dead cell apoptosis kit according to the manufacturer’s protocol. Green fluorescence from Alexa Fluor^®^ 488 Annexin V and red fluorescence from PI were detected using an Epics XL cytometer (*E*_x_ = 488 nm; *E*_m_ = 530 nm for Alexa Fluor^®^ 488 Annexin V and *E*_m_ = 575 nm for PI). Data were quantitatively analyzed with EXPO32 ADC software (Beckman Coulter, Brea, CA, USA). The cell population in the lower left quadrant (Annexin V^−^/PI^−^) represents live cells, the population in the lower right quadrant (Annexin V^+^/PI^−^) represents early apoptotic cells, and the population in the upper right quadrant (Annexin V^+^/PI^+^) represents late apoptotic or dead cells (Figure 5).

## Figures and Tables

**Table 1:** NMR spectroscopic data for compounds **1** and **2** in CD_3_OD (^1^H at 500 MHz, ^13^C at 125 MHz).

No.	1		2	
	*δ*_H_ (m, *J* in Hz)	*δ*_C_, Type	*δ*_H_ (m, *J* in Hz)	*δ*_C_, Type
1		176.4, C		176.8, C
3	4.37 (dd, 5.0, 9.8)	54.9, CH	3.85 (dd, 4.7, 9.1)	54.4, CH
4	2.59 (dd, 11.2, 11.2)	47.8, CH	2.54 (m)	45.8, CH
5	1.75 (m)	39.0, CH	2.05 (m)	40.1, CH
6		77.4, C		74.1, C
7	3.57 (d, 12.1)	76.7, CH	3.19 (d, 11.8)	73.6, CH
8	2.32 (m)	47.0, CH	2.67 (dd, 11.0, 11.0)	47.3, CH
9		66.7, C		65.7, C
10	3.10 (dd, 3.9, 14.8)	33.3, CH_2_	2.84 (dd, 4.8, 14.7)	33.4, CH_2_
	2.83 (dd, 5.3, 14.8)		3.00 (dd, 4.8, 14.7)	
11	1.17 (d, 6.9)	12.5, CH_3_	1.05 (d, 7.4)	13.5, CH_3_
12	1.17 (s)	22.3, CH_3_	1.24 (s)	24.8, CH_3_
13	5.81 (dd, 10.0, 15.0)	129.9, CH	5.98 (dd, 10.6, 15.7)	129.5, CH
14	5.15 (m)	134.5, CH	5.17 (m)	135.0, CH
15	2.03 (m)	41.9, CH_2_	2.44 (m)	42.0, CH_2_
	2.43 (d, 11.8)		2.00 (m)	
16	2.73 (m)	34.5, CH	2.78 (m)	34.6, CH
17	6.24 (d, 9.0)	149.8, CH	6.22 (d, 9.1)	150.0, CH
18		136.3, C		136.4, C
19		205.9, C		205.7, C
20	4.75 (dd, 6.2, 6.2)	72.3, CH	4.69 (dd, 5.5, 5.5)	72.5, CH
21	1.67 (m)	32.1, CH_2_	1.40 (m)	31.9, CH_2_
	1.37 (m)		1.64 (m)	
22	2.38 (m)	37.9, CH_2_	2.54 (m)	38.6, CH_2_
	1.67 (m)		1.86 (m)	
23		209.8, C		210.0, C
2′	7.11 (m)	125.6, CH	7.06 (m)	125.7, CH
3′		110.3, C		109.9, C
3′a		129.4, C		129.3, C
4′	7.58 (d, 7.9)	119.5, CH	7.53 (d, 7.9)	119.3, CH
5′	7.04 (dd, 7.9, 7.9)	120.1, CH	7.06 (m)	120.2, CH
6′	7.11 (m)	122.5, CH	7.12 (dd, 7.4, 7.4)	122.5, CH
7′	7.36 (d, 8.2)	112.4, CH	7.36 (d, 9.5)	112.6, CH
1′a		138.0, C		138.0, C
16-Me	1.03 (d, 6.7)	20.2, CH_3_	1.03 (d, 6.6)	20.2, CH_3_
18-Me	1.78 (s)	13.3, CH_3_	1.79 (s)	12.4, CH_3_

**Table 2:** Cytotoxic activity of compounds **1**–**9** ^a^.

Compounds	MDA-MB-231	LNCap	B16F10
**1**	>10	9.25 ± 0.80	>10
**2**	>10	>10	>10
**3**	>10	>10	7.15 ± 1.21
**4**	>10	2.93 ± 0.61	3.74 ± 0.32
**5**	>10	1.05 ± 0.24	2.10 ± 0.26
**6**	>10	0.62 ± 0.05	2.78 ± 0.15
**7**	>10	7.78 ± 1.80	4.67 ± 1.02
**8**	>10	3.29 ± 0.61	4.84 ± 0.75
**9**	>10	7.12 ± 2.02	>10
*cis*-platin	2.48 ± 0.74	1.04 ± 0.06	2.80 ± 0.11

^a^ Results are expressed as IC_50_ values ± SD in micromolar (μM), data were obtained from triplicate experiments, and *cis*-platin was used as positive control.

## Supplementary Materials

The following are available online at www.mdpi.com/1660-3397/14/12/233/s1, all 1D and 2D NMR, and HRESIMS spectra for compounds **1**–**2** are included.
