# Supplementary material for: Cytoglobosins H and I, New Antiproliferative Cytochalasans from Deep-Sea-Derived Fungus Chaetomium globosum
Source: Mar Drugs. 2016 Dec 20;14(12):233. doi: 10.3390/md14120233 (PMC5192470; doi:10.3390/md14120233)
Supplement: Supplementary file 1 [file marinedrugs-14-00233-s001.docx]

Supplementary Materials: Cytoglobosins H
and I, New Antiproliferative Cytochalasans from Deep-Sea-Derived Fungus *Chaetomium globosum*

Zhihan Zhang, Xitian Min, Junjun Huang, Yue Zhong, Yuehua Wu, Xiaoxia Li, Yinyue Deng, Zide Jiang, Zongze Shao, Lianhui Zhang and Fei He


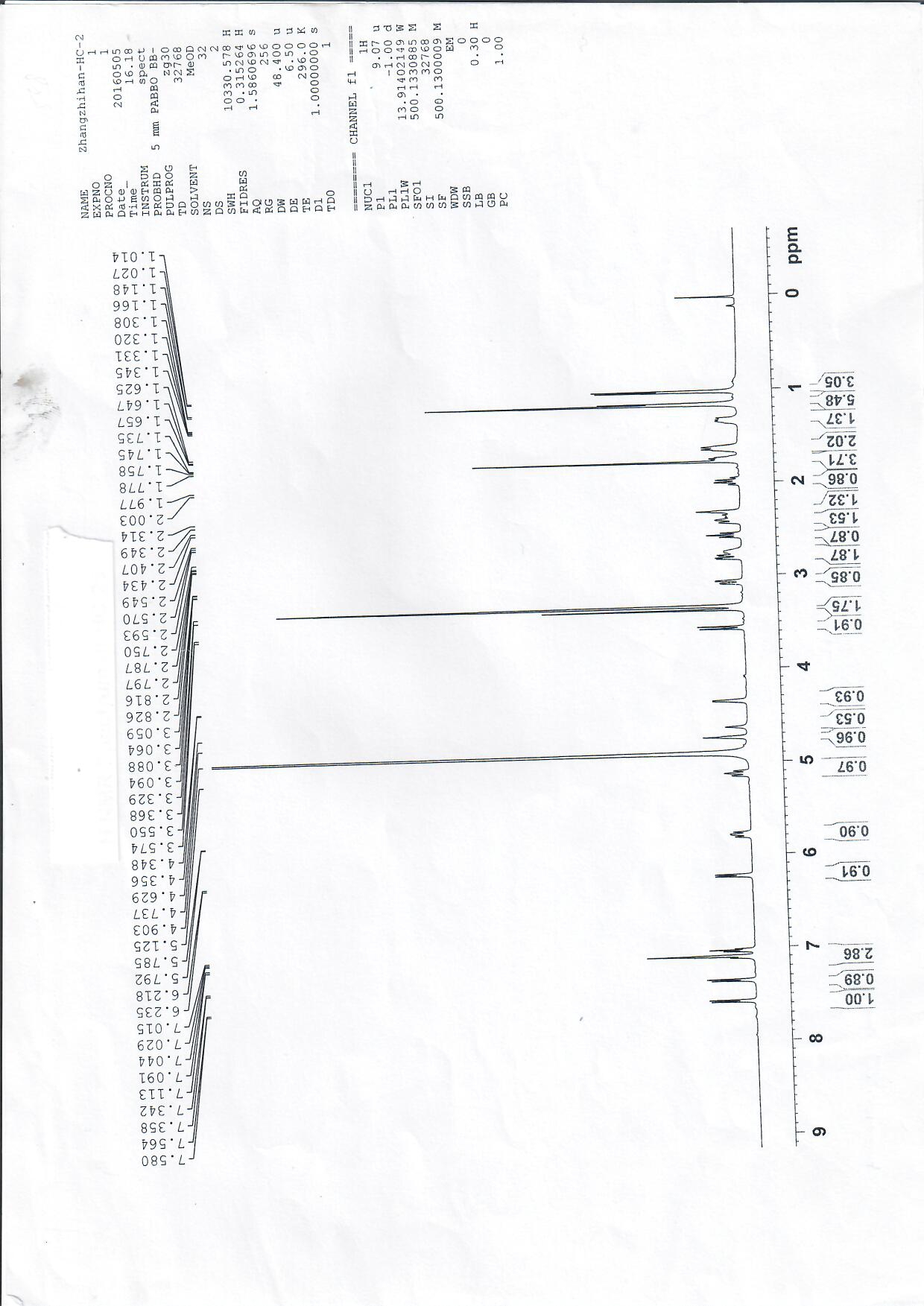


**Figure 1.** ^1^H NMR spectrum of cytoglobosins H (**1**).


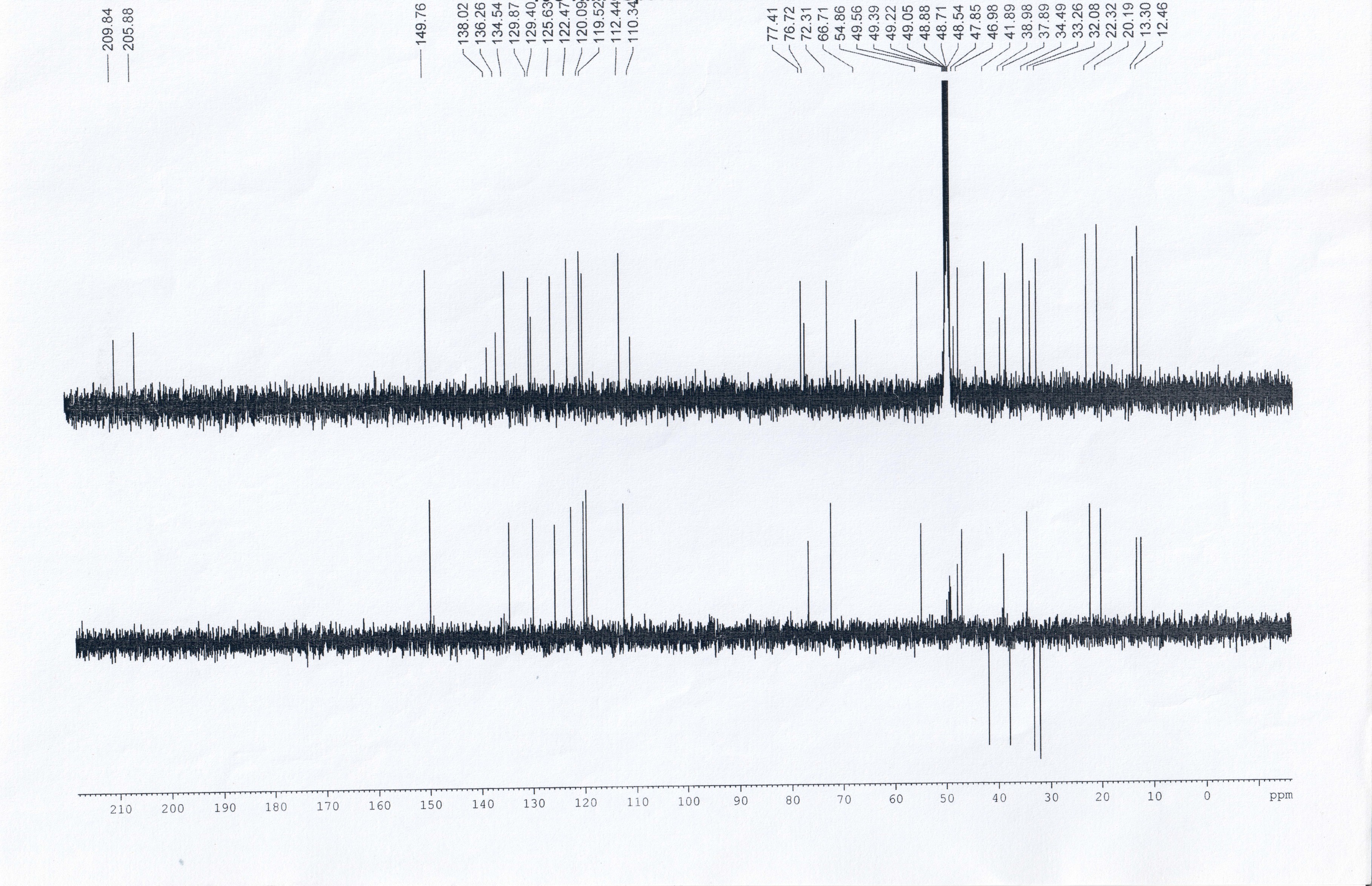


**Figure 2.** ^13^C NMR spectrum of cytoglobosins H (**1**).


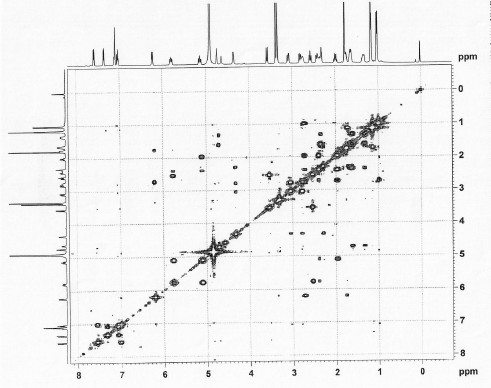


**Figure S3.** COSY spectrum of cytoglobosins H (**1**).


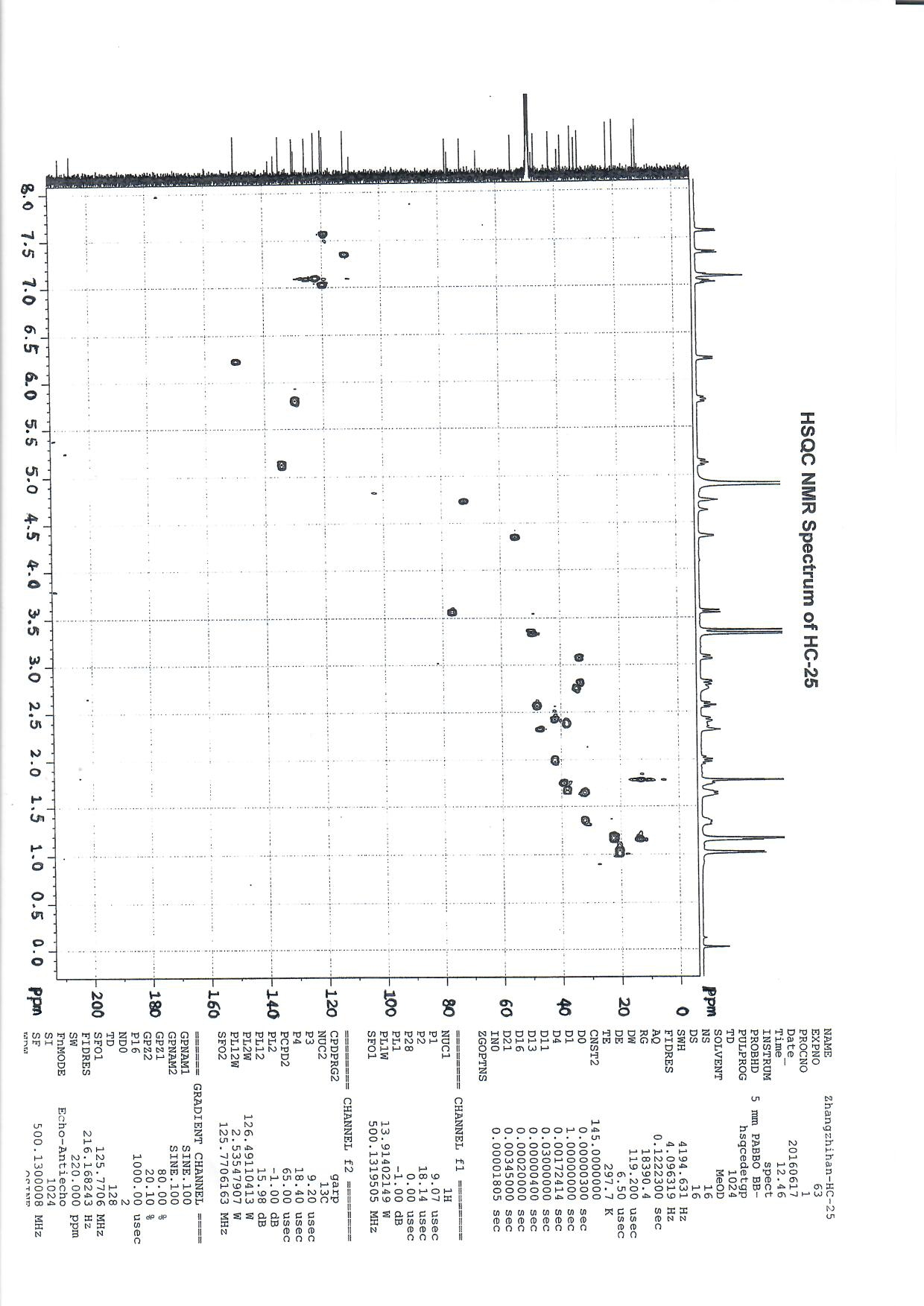


**Figure S4.** HSQC spectrum of cytoglobosins H (**1**)


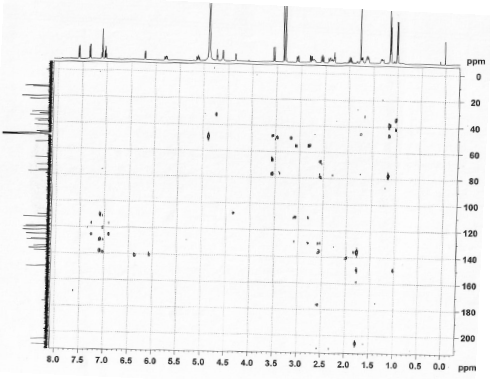


**Figure S5.** HMBC spectrum of cytoglobosins H (**1**)


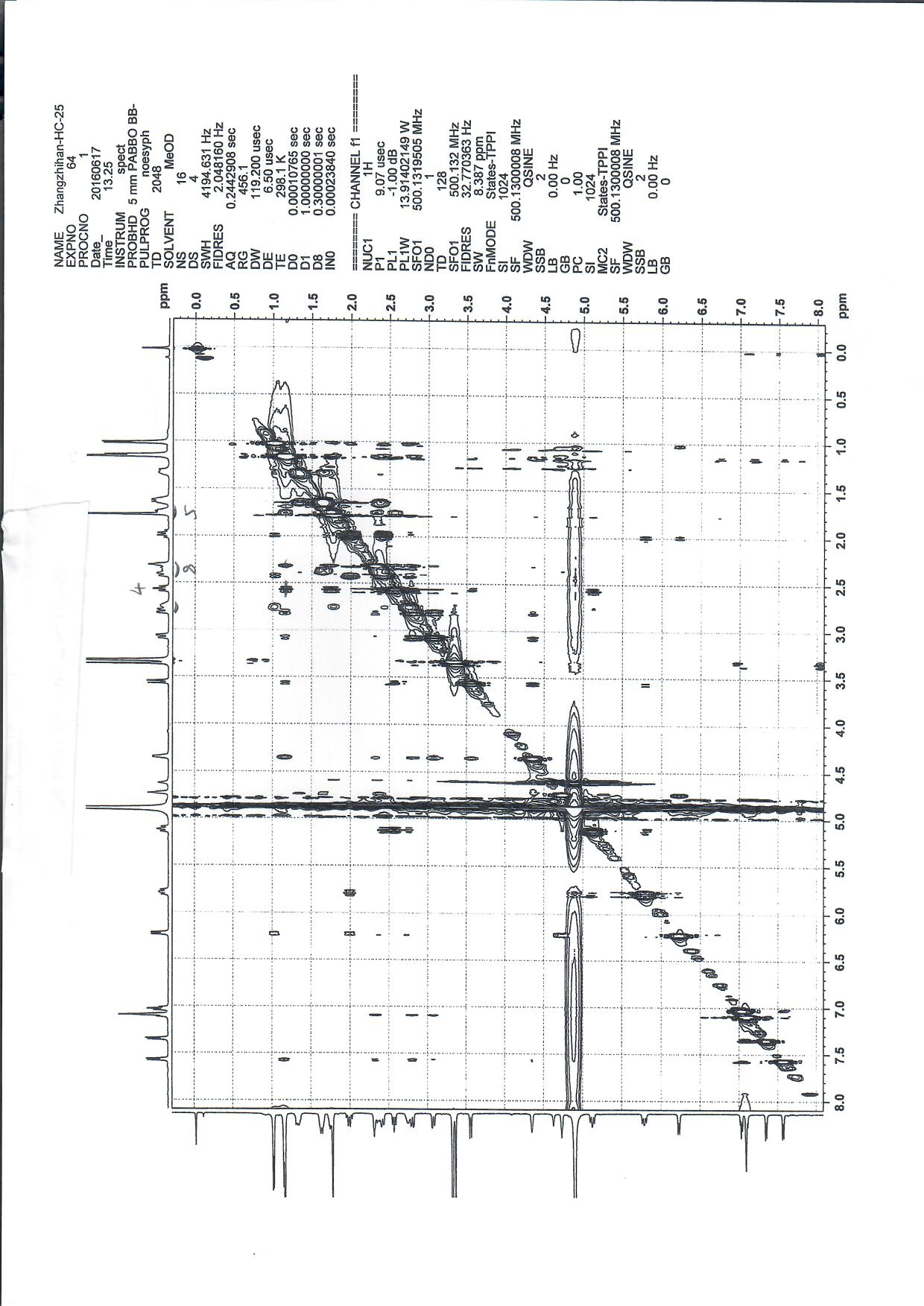


**Figure S6.** ROESY spectrum of cytoglobosins H (**1**).


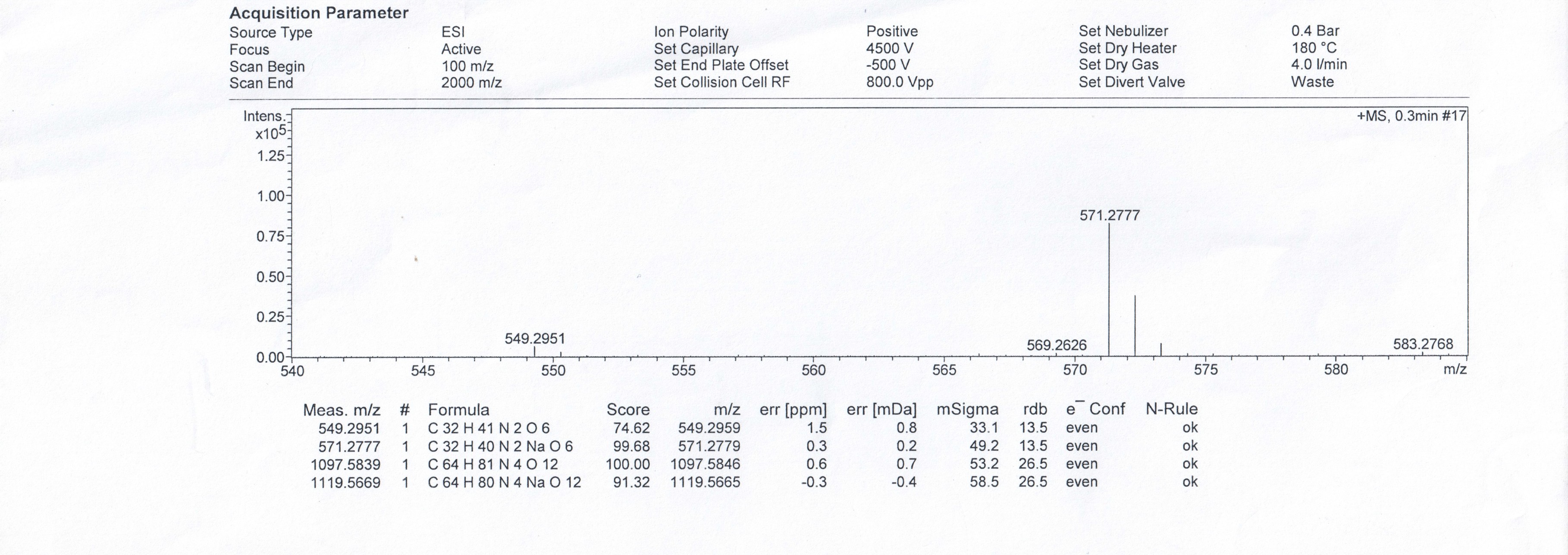


**Figure S7.** HRESIMS spectrum of cytoglobosins H (**1**).


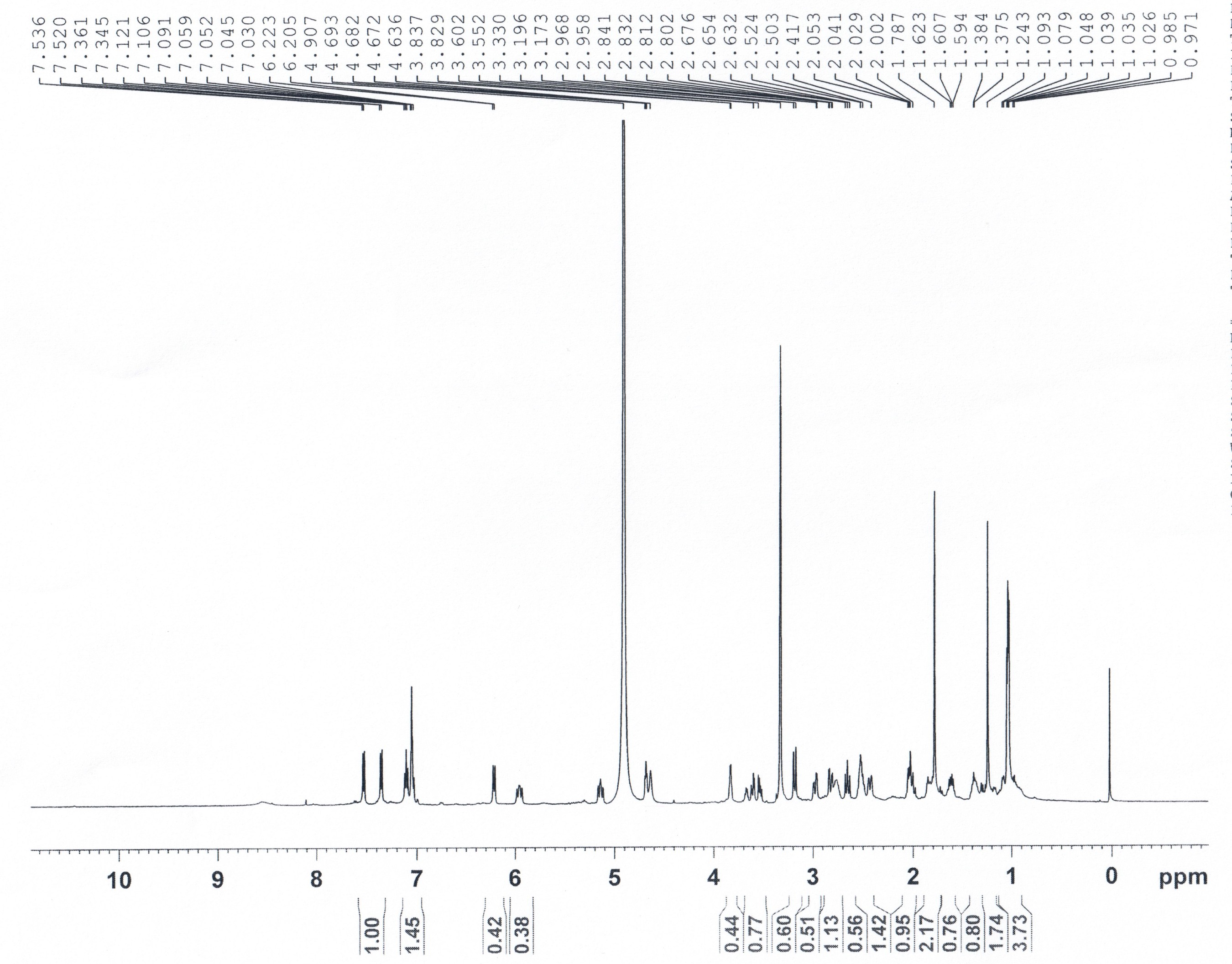


**Figure S8.** ^1^H NMR spectrum of cytoglobosins I (**2**).


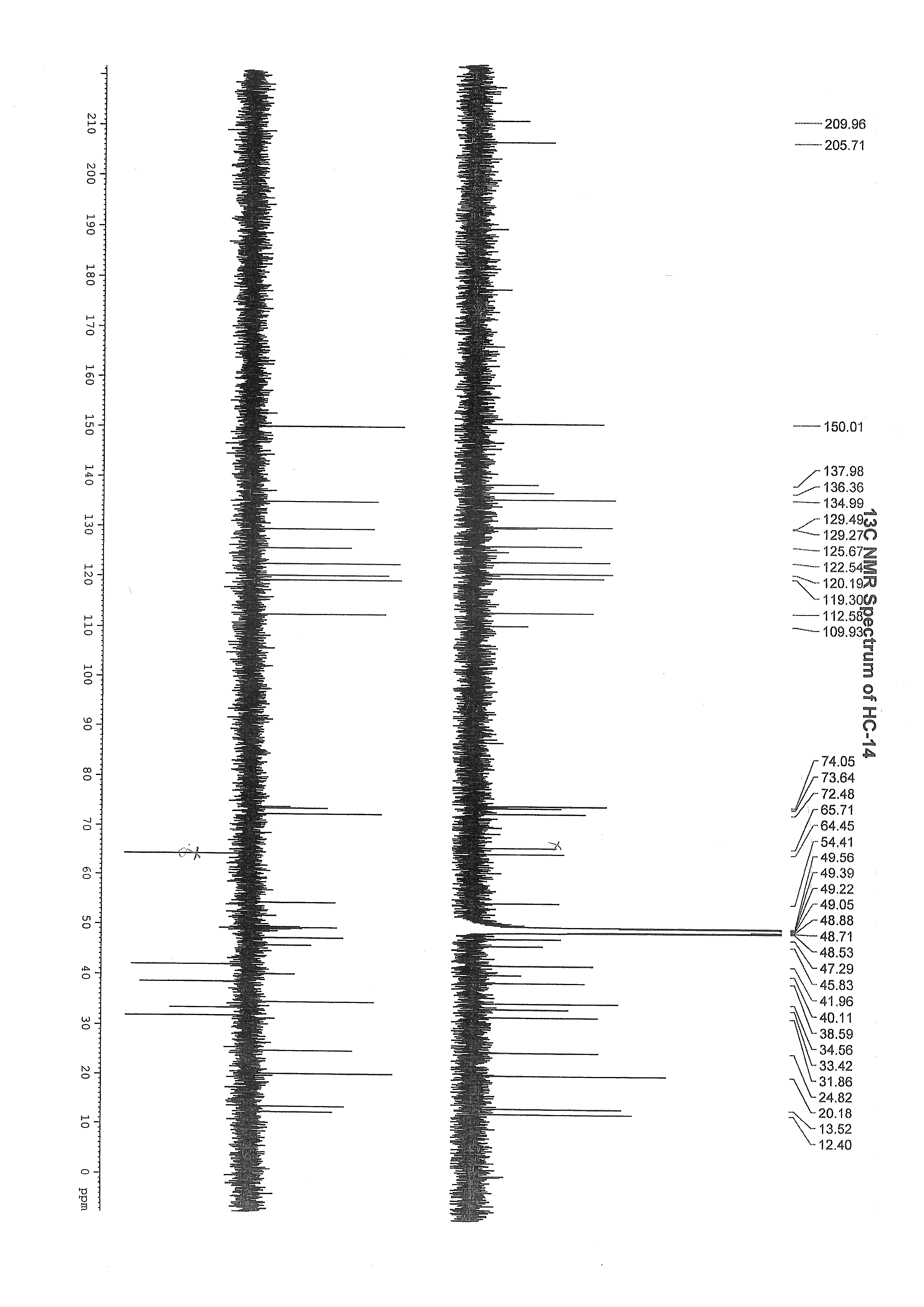


**Figure S9.** ^13^C NMR spectrum of cytoglobosins I (**2**).


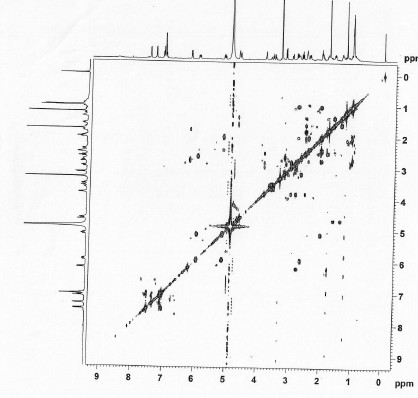


**Figure S10.** COSY spectrum of cytoglobosins I (**2**).


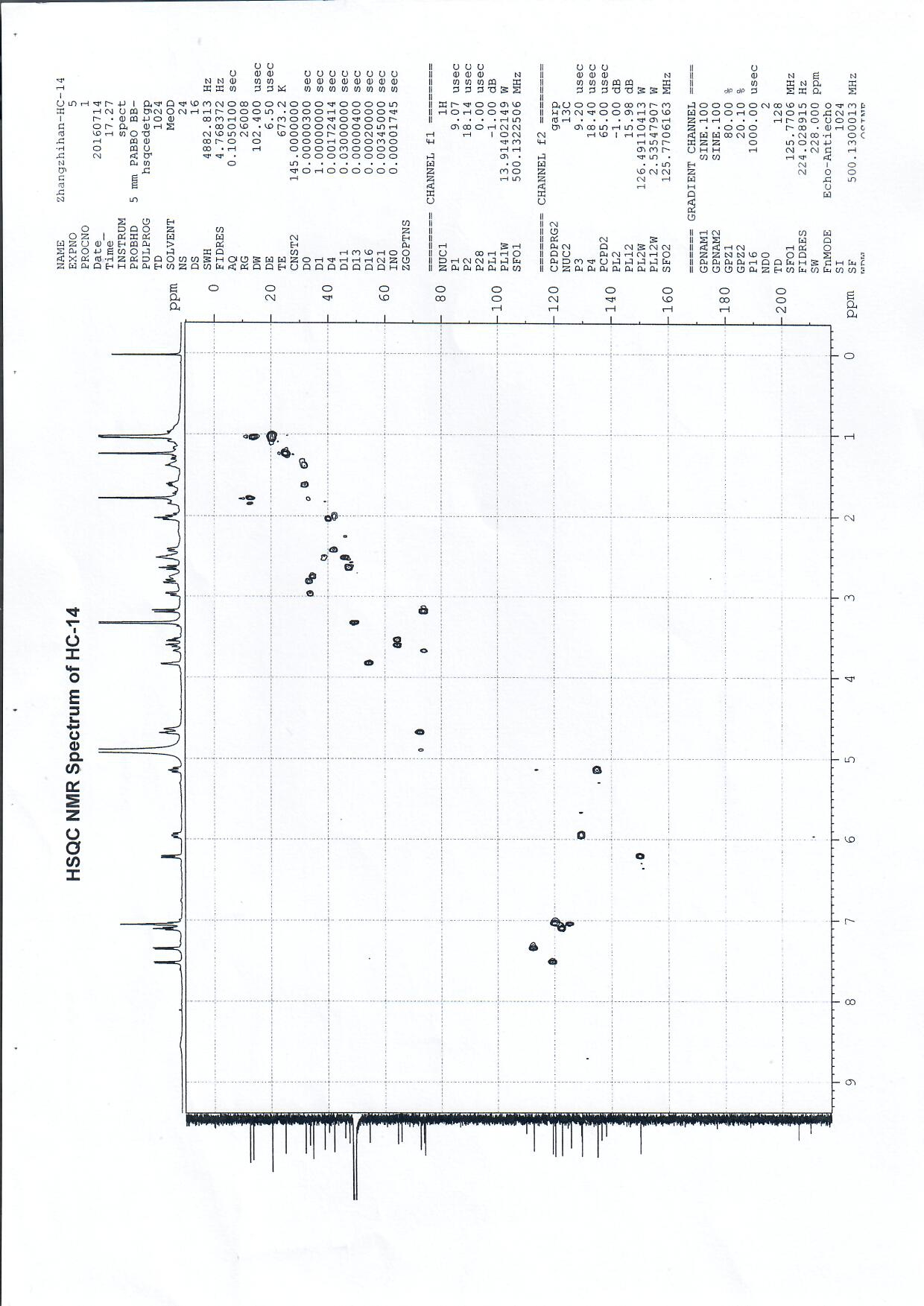


**Figure S11.** HSQC spectrum of cytoglobosins I (**2**).


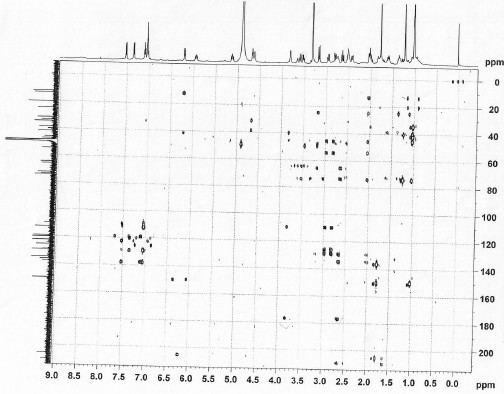


**Figure S12.** HMBC spectrum of cytoglobosins I (**2**).


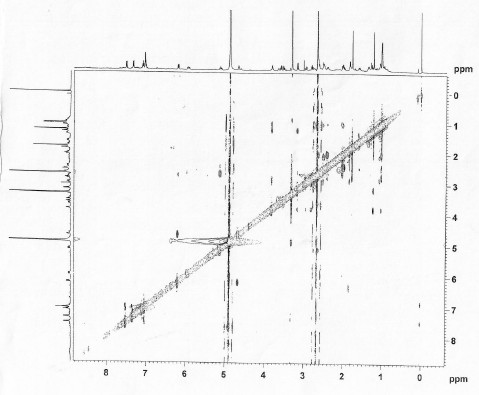


**Figure S13.** ROESY spectrum of cytoglobosins I (**2**).


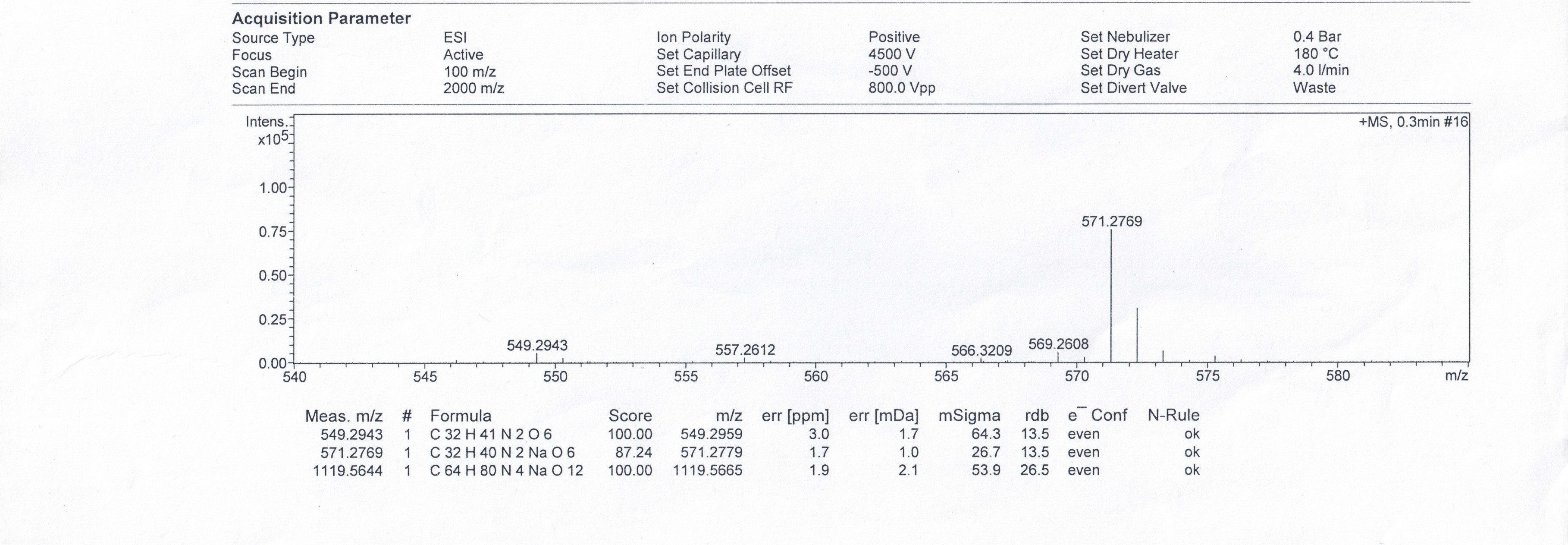


**Figure S14.** HRESIMS spectrum of cytoglobosins I (**2**).


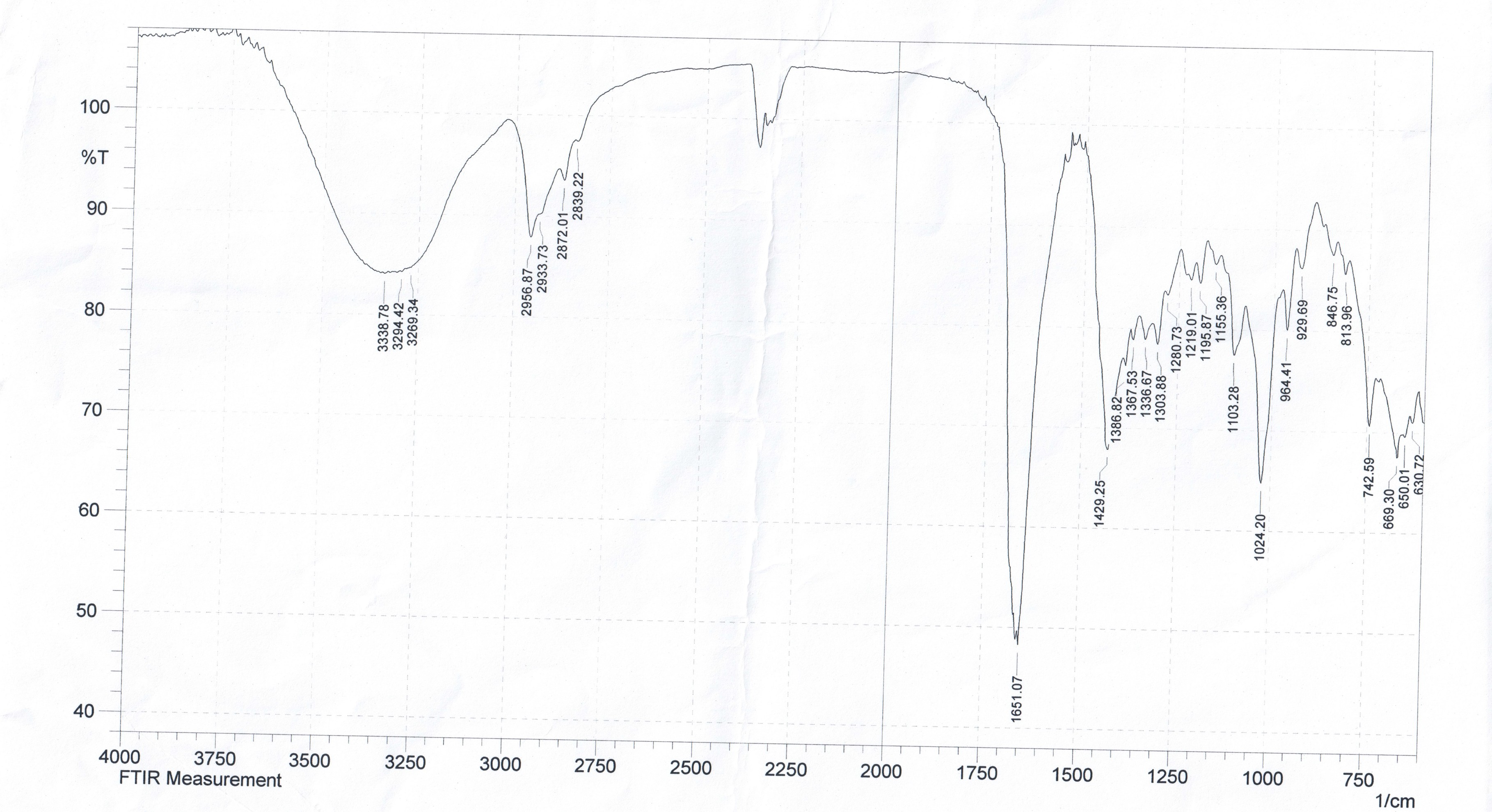


**Figure S15.** IR spectrum of cytoglobosins H (**1**).


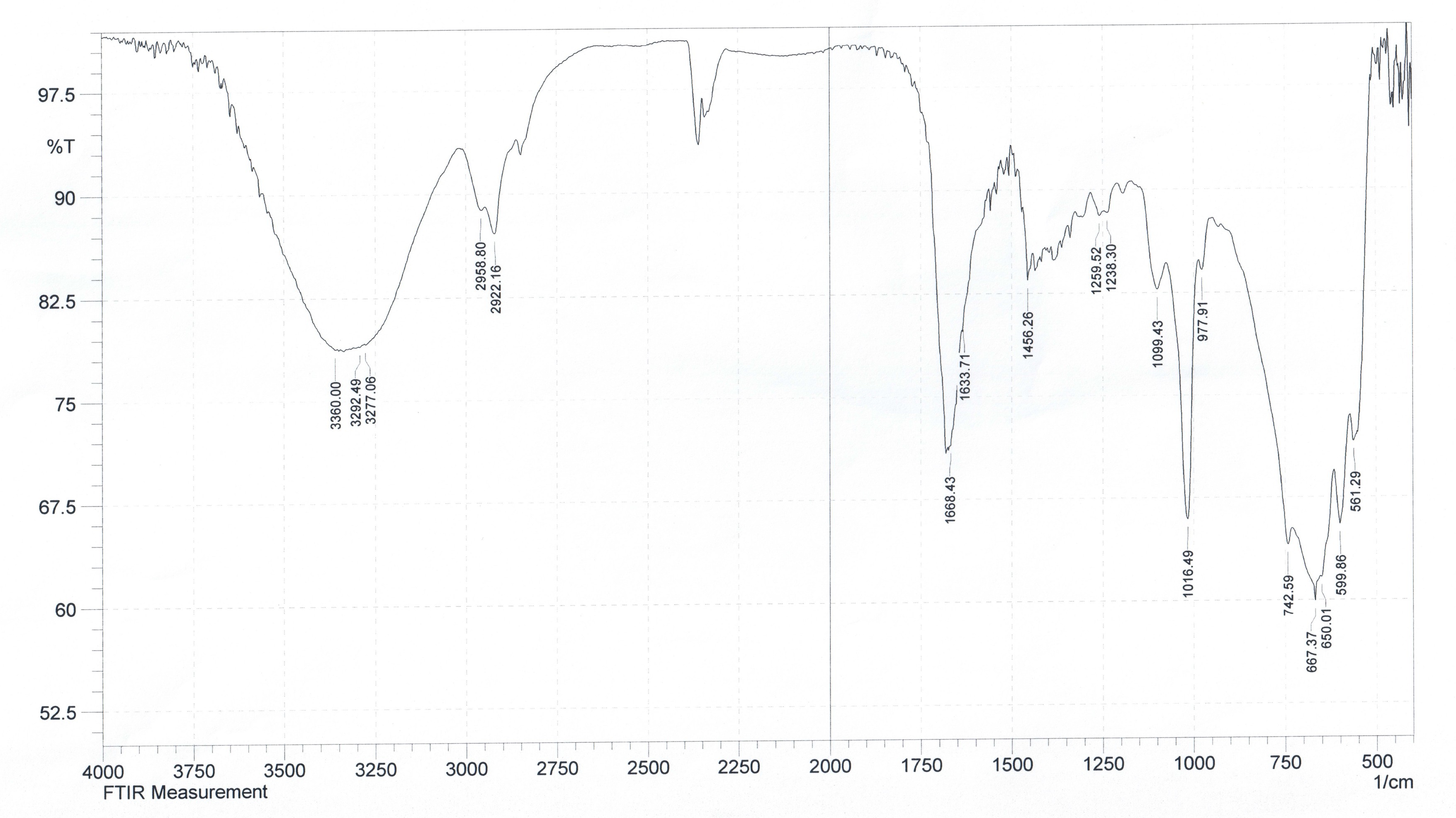


**Figure S16.** IR spectrum of cytoglobosins I (**2**).


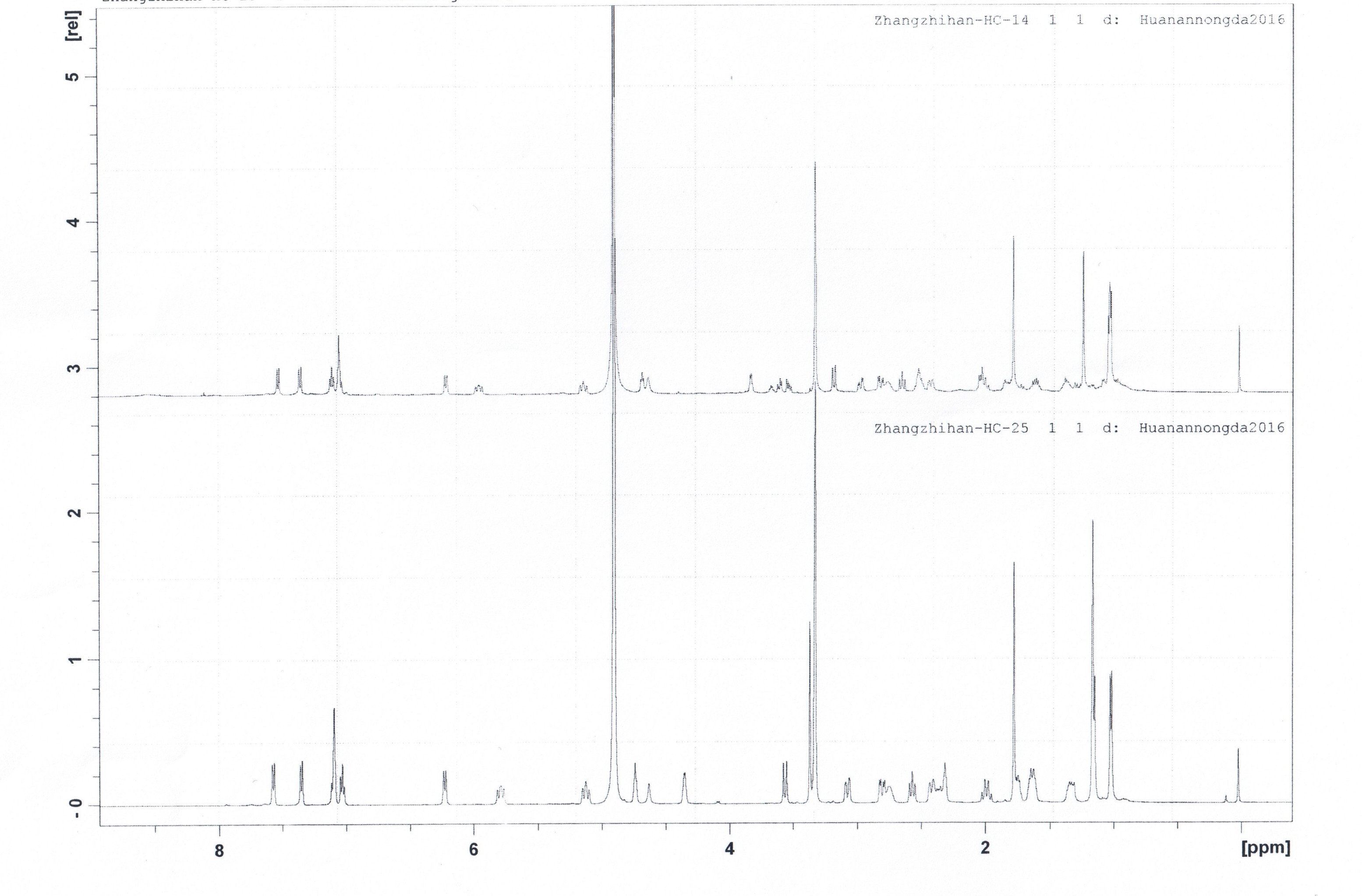

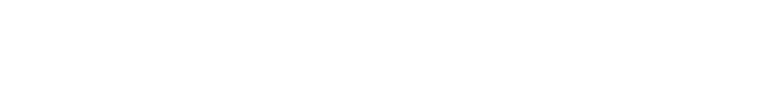

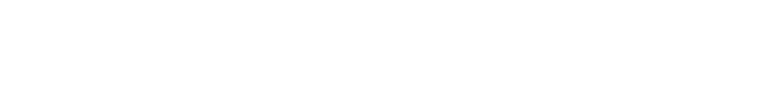


Compound **1**

Compound **2**

**Figure S17.** Comparison of ^1^H spectra between **1** and **2**.
